# Research Progress of TXNIP as a Tumor Suppressor Gene Participating in the Metabolic Reprogramming and Oxidative Stress of Cancer Cells in Various Cancers

**DOI:** 10.3389/fonc.2020.568574

**Published:** 2020-10-21

**Authors:** Yiting Chen, Jieling Ning, Wenjie Cao, Shuanglian Wang, Tao Du, Jiahui Jiang, Xueping Feng, Bin Zhang

**Affiliations:** ^1^Department of Oncology and Institute of Medical Sciences, National Clinical Research Center for Geriatric Disorders, Xiangya Hospital, Central South University, Changsha, China; ^2^Department of Histology and Embryology, Xiangya School of Medicine, Central South University, Changsha, China; ^3^Institute of Medical Sciences, Xiangya Hospital, Central South University, Changsha, China

**Keywords:** TXNIP (thioredoxin interacting protein), cancer, oxidative stress, research progress, clinical significance

## Abstract

Thioredoxin-interacting protein (TXNIP) is a thioredoxin-binding protein that can mediate oxidative stress, inhibit cell proliferation, and induce apoptosis by inhibiting the function of the thioredoxin system. TXNIP is important because of its wide range of functions in cardiovascular diseases, neurodegenerative diseases, cancer, diabetes, and other diseases. Increasing evidence has shown that TXNIP expression is low in tumors and that it may act as a tumor suppressor in various cancer types such as hepatocarcinoma, breast cancer, and lung cancer. TXNIP is known to inhibit the proliferation of breast cancer cells by affecting metabolic reprogramming and can affect the invasion and migration of breast cancer cells through the TXNIP-HIF1α-TWIST signaling axis. TXNIP can also prevent the occurrence of bladder cancer by inhibiting the activation of ERK, which inhibits apoptosis in bladder cancer cells. In this review, we find that TXNIP can be regulated by binding to transcription factors or other binding proteins and can also be downregulated by epigenetic changes or miRNA. In addition, we also summarize emerging insights on TXNIP expression and its functional role in different kinds of cancers, as well as clarify its participation in metabolic reprogramming and oxidative stress in cancer cells, wherein it acts as a putative tumor suppressor gene to inhibit the proliferation, invasion, and migration of different tumor cells as well as promote apoptosis in these cells. TXNIP may therefore be of basic and clinical significance for finding novel molecular targets that can facilitate the diagnosis and treatment of malignant tumors.

## Introduction

Cancers are chronic diseases that pose serious threats to human health and have increasing incidence rates worldwide. The burden of cancers is increasing relentlessly due to their huge economic and human costs. At present, there are many treatments for malignant tumors, such as surgery, chemotherapy, radiation therapy, and immunotherapy. However, although great progress has been achieved in the treatment of malignant tumors, the precise cause of the disease is poorly understood, which has become a major limiting factor in improving the prognosis of patients.

As a result of increased research on genomics and epigenetics, it is now anticipated that gene-targeted therapies may be effective in preventing the occurrence and development of human malignant tumors. Therefore, the search for new target genes is of particular significance for the development of cancer treatments. Thioredoxin-interacting protein (TXNIP), a 50-kDa protein (1), is also known as thioredoxin-binding protein 2 (TBP-2) (2) or vitamin D3 upregulated protein 1 (VDUP-1) (3). The gene encoding TXNIP is located in regions 1q21–22 of human chromosome 1 and is about 4,174 bp in length, containing 8 exons (4). The TXNIP gene is normally found in the nucleus but can shuttle into the mitochondria under certain conditions (5). TXNIP was first identified in 1995 as a gene that is upregulated in HL-60 cells treated with vitamin D3 (6). As a negative regulator of thioredoxin (TRX) (7), a major redox-regulating molecule, TXNIP participates in regulating the cellular redox status. Oxidative stress caused by the excessive production or accumulation of reactive oxygen species (ROS) is known to be related to cancer pathology and cancer treatment. TXNIP is important for regulating mitochondrial function, inducing apoptosis, and inhibiting growth and metastasis; in addition, it also plays roles in the development of natural killer cells, promoting cell-cycle arrest and regulating glucose metabolism and inflammatory signaling (8–10). TXNIP belongs to the α-arrestin protein family (11), the members of which have two domains, namely, SH3 and PPxY (12); through these domains, TXNIP can interact with other proteins to perform different biological roles.

## Cancers of Endocrine Glands and Genital Tract (Table 1)

### TXNIP and Breast Cancer

**Figure 1:**
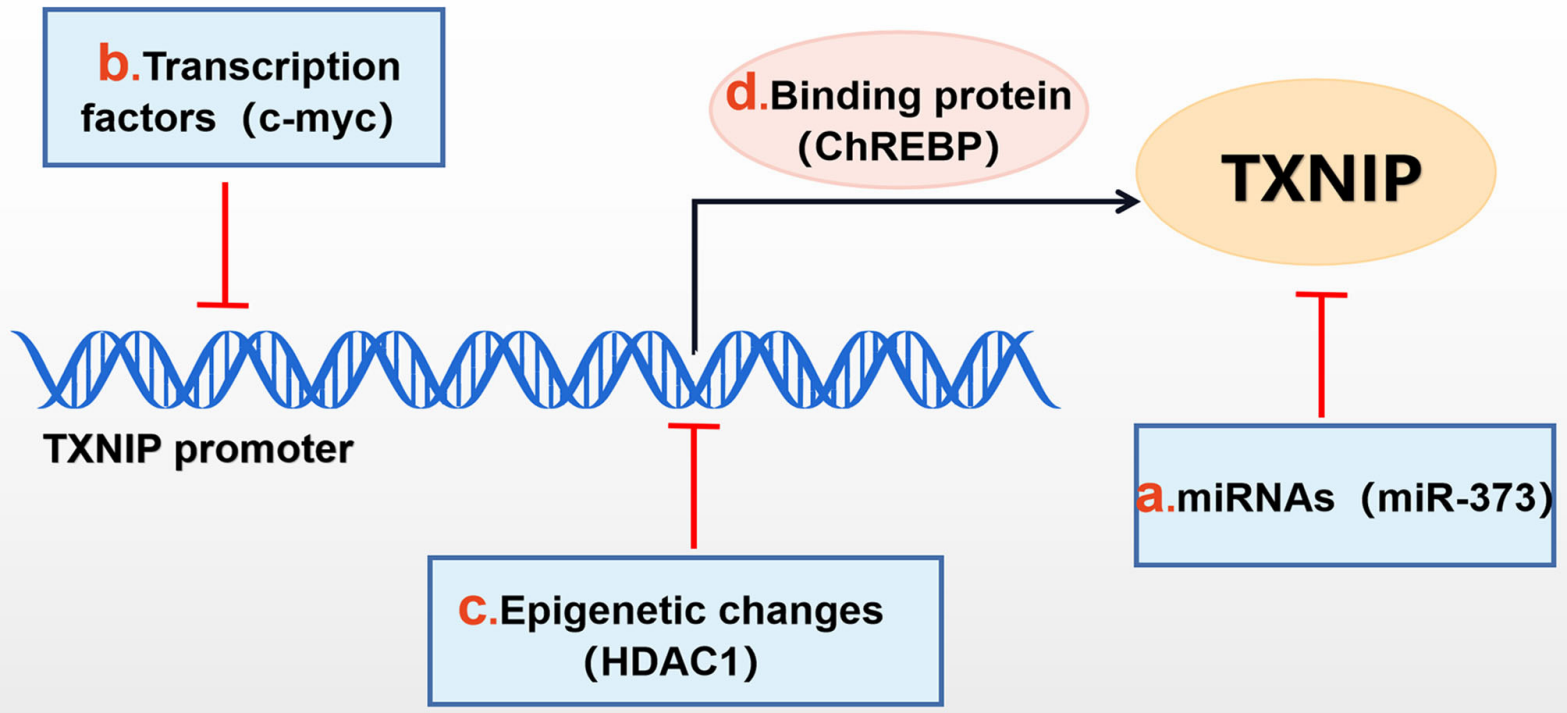
Mechanisms of TXNIP regulation. TXNIP expression can be negatively affected by **(b)** transcription factors (c-myc,et), **(c)** epigenetic changes [histone deacetylase 1 (HDAC1), et], **(a)** miRNAs (miR-373, et) and can be positively affected by **(d)** binding proteins, such as ChREBP.

**Figure 2:**
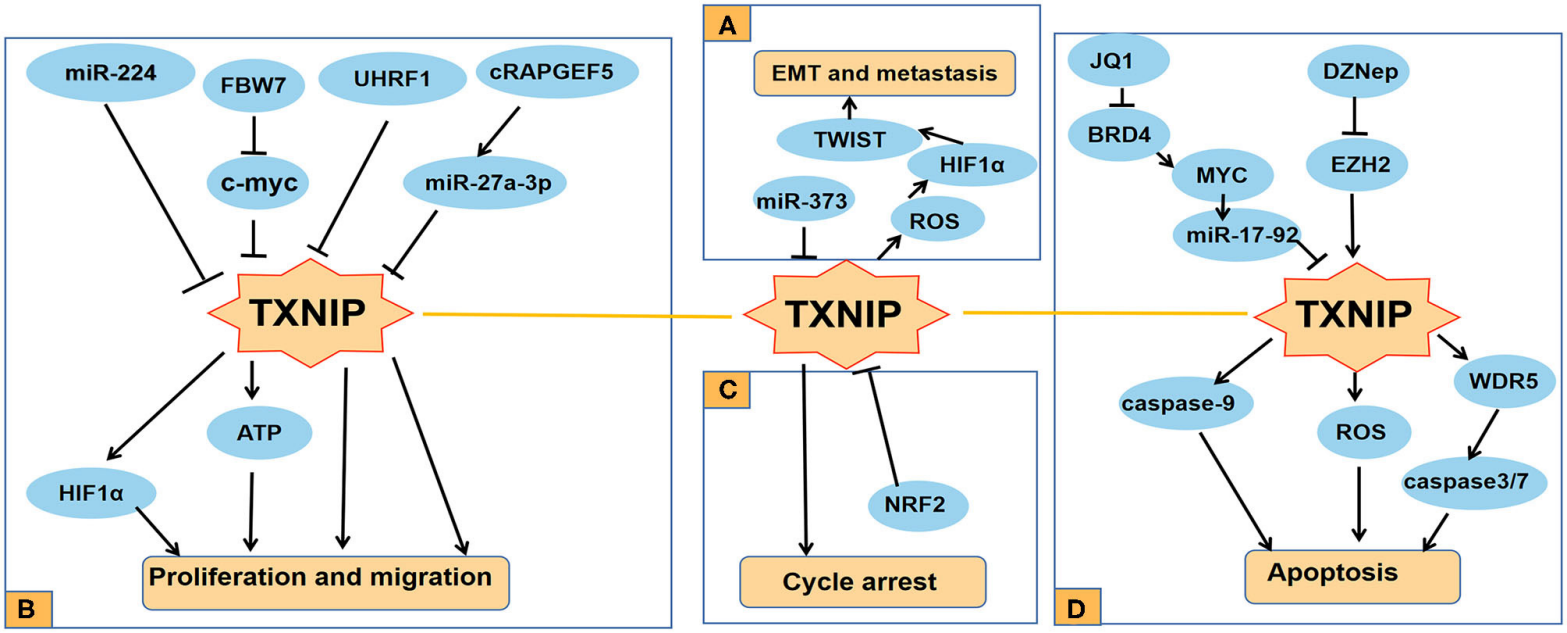
Functions of TXNIP involved in several typical cancers. **(A)** TXNIP participates in different signaling pathways to inhibit the proliferation and migration of PDAC, RC, and other cancer cells; **(B)** miR-373 drives the transformation and metastasis of breast cancer by the TXNIP/HIF1α/TWIST signaling axis; **(C)** Knocking down NRF2 can promote the cycle arrest of PCA cells by increasing the expression level of TXNIP; **(D)** TXNIP participates in different signaling pathways to promote the apoptosis of cancer cells such as AML and lung cancer.

Breast cancer arises from breast epithelial tissue. Because TXNIP is an effective tumor suppressor, it is often found to be expressed at low levels in different kinds of tumors (25); in particular, TXNIP expression levels in human breast cancer MCF-7 cells are markedly decreased (26, 27). It has been well-documented that TXNIP expression is positively associated with the loss of tumor differentiation and that TXNIP levels gradually decrease as tumors advance in animal models. TXNIP levels have been found to recover immediately after treatment with histone deacetylase inhibitors, suggesting that histone deacetylation may influence TXNIP expression. *TXNIP* gene knockout can promote the proliferation of breast cancer cells, which is accompanied by decreased p27 expression and increased glucose transporter type 1 (GLUT1) levels (13). Most cases of mortality due to breast cancer involve distant metastases (28). The epithelial to mesenchymal transition (EMT) is a key process promoting tumor metastasis, wherein the levels of the adhesion protein E-cadherin decrease (29). Hypoxia-inducible factor 1α (HIF-1α) is involved in the metastasis and progression of various cancers and is closely related to the failure of various cancer treatments and the resulting patient mortality (30). TWIST and HIF-1α are two key inducers of EMT in cancer cells, with the former being a downstream effector of HIF-1α. The oncogene miR-373 is known to promote tumor migration and invasion in breast cancer, although the mechanism is poorly understood (31–34). It has, however, been shown that miR-373 decreases the expression of TXNIP by interacting with the 3′ untranslated region (UTR) of TXNIP (Figure 1a) to reduce TXNIP-dependent ROS, activate EMT, and thereby promote tumor migration and invasion. It has been postulated that the activation of the miR-373-TXNIP-HIF1α-TWIST signaling axis is related to the poor prognosis of breast cancer patients (Figure 2A) and that TXNIP levels may be a potential prognostic biomarker for breast cancer (14). Patients with triple negative breast cancer (TNBC) have lower survival rates, higher rates of recurrence, and greater changes of metastasis than those with other types of breast cancer. Previous studies have proven that the transcription factor c-myc drives glucose metabolism in TNBCs. One possible mechanism of this process is that c-myc competes with the related transcription factor MondoA to interact with the E-box-containing region in the TXNIP promoter, so as to reduce the expression of TXNIP (Figure 1b). This results in an enhancement of aerobic glycolysis in the TNBC cells, thereby providing more ATP to fuel the growth and proliferation of TNBC cells, making the treatment of TNBC more difficult. Researchers have also shown that low level of TXNIP expression is related to poor prognosis in patients with breast cancer, and so, TXNIP may be of great importance in providing new targets for the treatment of patients with TNBC (15).

**Table 1:** TXNIP expression in cancers of the endocrine glands and genital tracts.

**Tumor type**	**Upstream or downstream molecular**	**Significance**	**TXNIP expression**	**Samples**	**References**
Breast cancer	p27 and GLUT 1	Promote the growth of breast cancer cells *in vitro* and *in vivo*	Down	Cell lines, tissue	(13)
Breast cancer	miR-373 and HIF1α	miR-373 drives the transformation and metastasis of breast cancer	Down	Cell lines, tissue, animal	(14)
Breast cancer	c-Myc	C-myc competes with related transcription factor MondoA and drives glucose metabolism	Down	Cell lines	(15)
Thyroid cancer	–	Expression correlates with metastatic properties	Down	Cell lines, animal	(16)
PTC	–	The expression of TXNIP in PTC tissues was lower than that in normal thyroid tissues	Down	tissue	(17)
Renal cancer	UHRF1	UHRF1 can recruit HDAC1 to the TXNIP's promoter and mediate the deacetylation of histone H3K9	Down	Cell lines, tissue	(18)
Renal cancer	cRAPGEF5	cRAPGEF5 targets miR-27a-3p to promote the proliferation and migration of RCC	Down	Cell lines, tissue	(19)
Bladder cancer	ERK	Improve disease-specific survival	Down	Cell lines, tissue, animal	(20)
Prostate cancer	c-Myc and GLS 1	C-myc activates glutamine 1 (GLS 1) to accelerate the proliferation	Down	Cell lines, tissue	(21)
Prostate cancer	NRF2	RNF2 binds to TXNIP's promoter to increase apoptosis and inhibit proliferation	Down	Cell lines, tissue, animal	(22)
Cervical cancer	MondoA	MondoA overexpression inhibited cell proliferation, migration, and invasion	Down	Cell lines	(23)
Endometrial cancer	Vitamin D3	VitaminD3 can increase the expression of TXNIP to inhibit the proliferation of endometrial cancer cells	Down	Cell lines	(24)

*p27, tumor protein 27; GLUT1, Glucose transporter type 1; HIF1α, hypoxia-inducible factor-1α; UHRF1, ubiquitin-like ringfinger domains 1; cRAPGEF5, CircRNA RAPGEF5; ERK, extracellular regulated protein kinases; GLS 1, Glutaminase 1; RNF2, nuclear factor E2-related factor 2*.

### TXNIP and Thyroid Cancer

Thyroid cancer arises from the follicles or thyroid cells adjacent to the follicles. Despite the decrease in thyroid cancer-related morbidity in recent decades (35), the incidence rate of thyroid cancer is increasing rapidly and thyroid cancer represents a considerable medical burden (36–38). Surgery, radiation therapy, and chemotherapy are common treatments for thyroid cancer; however, the prognosis for patients with thyroid cancer remains poor (39, 40). It has been shown that TXNIP overexpression decreases the growth of HTh74 cells and inhibits glucose uptake. It has also been shown that TXNIP overexpression in T238 cells inhibits tumor growth and decreases metastasis in a mouse model of *in situ* thyroid cancer (16). Papillary thyroid cancer (PTC), which causes serious harm to human health, accounts for 80–90% of all thyroid cancers (41), and its incidence rate is increasing year by year. The pathogenesis of PTC is related to the dysfunction of many oncogenes, apoptosis-related genes, and tumor suppressor genes. With the development of proteomics and genomics and the continuous progress of research on thyroid cancer, researchers have found that TXNIP is closely related to PTC (42). TXNIP expression is lower in PTC tissues compared to normal thyroid tissue. The low expression levels of TXNIP are also related to the incidence of lymph node metastasis in PTC; however, they are unrelated to the gender or age of patients (17).

### TXNIP and Renal Cancer

Renal cancer (RC) is a type of cancer that originates in the nephron (43). The incidence of renal cell carcinoma (RCC) is also increasing year by year (44). Studies have confirmed that about 20–30% patients with RC have distant metastasis at the time of primary diagnosis (45). Bioinformatics analyses have shown that the expression of TXNIP in clear cell renal cell carcinoma (CCRCC) is lower than in normal tissues. Furthermore, the prognosis of patients with CCRCC who have low levels of TXNIP expression is worse than that of patients with high TXNIP expression levels. In addition, the Wnt, mitogen-activated protein kinase (MAPK), phosphatidylinositol, and transforming growth factor-β (TGF-β) signaling pathways as well as the autophagy signaling pathway may be the key pathways regulated by TXNIP in patients with CCRCC (46). Ubiquitin-like with PHD and Ring Finger Domains 1 (UHRF1) is an important epigenetic regulator that belongs to the UHRF family. In RCC, UHRF1 can recruit histone deacetylase 1 (HDAC1) to the TXNIP promoter (Figure 1c), which decreases TXNIP expression and promotes the occurrence and development of RCC (Figure 2B) (18). It has also been reported that circular RNA (cirRNA) plays a key role in the development of cancer. CirRNA RAPGEF5 (cRAPGEF5) is a circular RNA derived from exons 2–6 of the *RAPGEF5* gene. Studies have shown that cRAPGEF5 targets miR-27a-3p to upregulate its expression. miR-27a-3p targets the 3′UTR in TXNIP to downregulate TXNIP expression, thereby promoting the tumor proliferation and migration in RCC (Figure 2B). TXNIP may therefore be a promising prognostic biomarker for RCC (19).

### TXNIP and Bladder Cancer

Bladder cancer (BC) is the most common malignant tumor of the urinary tract (47). About 70% of patients diagnosed with bladder cancer have non-muscle-infiltrating BC, with the remaining 25–30% having myometrial-infiltrating BC. The 5-year survival rate for patients with metastatic BC is only about 15% (48). Although cisplatin-based chemotherapy benefits patients with advanced and metastatic BC in neoadjuvant treatment, the adverse reactions are significant (49). In neoadjuvant tumors, high levels of TXNIP are considered to be an independent marker for improving disease-specific survival (50). In this regard, it has been shown that TXNIP levels are reduced in BC (51, 52). TXNIP can also negatively regulate the occurrence of bladder cancer by inhibiting the activation of ERK, which is induced by stromal cell-derived factor-1/C-X-C chemokine receptor type 4 signaling (20). This signal transduction pathway could therefore be an effective target for the prevention or treatment of BC.

### TXNIP and Prostate Cancer

Prostate cancer (PCA) is an epithelial malignant tumor which is the second leading cause of cancer-related death in men (53). Patients with early PCA can adopt radical treatment. In contrast, hormone-sensitive patients with advanced PCA are often treated with endocrine therapy, although it is often difficult to achieve a clinical cure. Therefore, finding new therapeutic targets for the treatment of PCA is particularly urgent. Studies have shown that the consumption of glycolytic intermediates leads to a continuous decline in TXNIP expression caused by 1,25(OH)_2_D_3_ in prostate cells, consistent with the activation of AMP-activated protein kinase (AMPK) signaling and the decrease in c-myc expression (54). Metabolic reprogramming induced by the proto-oncogene c-myc triggers the dependence of cells on exogenous glucose and glutamine. C-myc activates glutaminase 1 (GLS1) and reduces the activity of the transcription factor MondoA to downregulate TXNIP expression, so as to accelerate the proliferation of PCA cells (21). In human PCA cells, increased glucose metabolism dependently or independently induces the transcription of the TXNIP gene and thereby the expression of TXNIP. Glucose-induced TXNIP requires a glucose regulatory system, including the carbohydrate response element-binding protein (ChREBP) and the carbohydrate response element (ChoRE) (55). It has been shown that nuclear factor E2-related factor 2 (NRF2) is often overexpressed in different human cancers and that it is significantly related to a shortened overall survival time, suggesting that NRF2 may be a novel prognostic biomarker (56). It has also been reported that the NRF2 expression is high in PCA and that knocking down NRF2 can promote TXNIP expression by binding to the TXNIP promoter. This can cause cell-cycle arrest in PCA cells, increasing apoptosis and inhibiting the proliferation of tumor cells (Figure 2C) (22).

### TXNIP and Cervical Cancer

Cervical cancer is one of the most common gynecological malignancies (57). The pathogenesis of cervical cancer is a complex process involving multiple factors and steps (58, 59). It is therefore important to search for molecular markers and therapeutic targets for the treatment of cervical cancer. Research has shown that TXNIP overexpression inhibits cell proliferation, invasion, and migration in HeLa cells, whereas TXNIP silencing has the opposite effect in c-33a cells. Moreover, MondoA, rather than ChREBP overexpression, can inhibit cell invasion and migration by upregulating TXNIP in HeLa cells (23).

### TXNIP and Endometrial Cancer

Endometrial cancer is the fourth most common cancer among women worldwide (60–62). Most patients have a good prognosis in the early stage of the disease after treatment (63, 64). Although the prognosis of early treatment is good, it is still important to find novel therapeutic molecular targets for its treatment. Studies have confirmed that vitamin D3 plays a key role in the prevention and treatment of various cancers (24), and the concentration of vitamin D in the blood is also considered as a potential prognostic indicator (65–67). It has been shown that vitamin D3 can regulate the levels of ROS in endometrial cancer cells by increasing TXNIP expression resulting in an inhibition of human endometrial cancer cell proliferation. Vitamin D3 may therefore be important in the treatment of endometrial cancer (68).

## Cancers of the Digestive System (Table 2)

### TXNIP and Liver Cancer

Liver cancer is a disease of the digestive system. Primary liver cancer originates from the epithelial or mesenchymal tissue in the liver (76). Some studies have shown that TXNIP expression levels in hepatoma cell lines are low or absent. As discussed earlier, it is known that vitamin D3 can stimulate TXNIP expression, resulting in reduced cell proliferation and increased apoptosis. The expression of TXNIP in hepatocytes can cause oxidative damage. Stimulating the expression of TXNIP through vitamin D3 and other factors can reduce the canceration of chronic survival patients (69). There are two binding sites, for ChoRE-a and ChoRE-b, in the TXNIP promoter. At high sugar concentrations, ChREBP can bind to the upstream promoter region of TXNIP and promote TXNIP expression (Figure 1d). It has been found that heparin influences cell growth, differentiation, invasion, and migration and promotes the transcription of TXNIP in hepatoma cells by binding to the ChoRE-b site (70). Studies have also shown that chronic hepatitis B virus (HBV) infection is related to the occurrence and development of hepatocellular carcinoma (HCC) (77). It is well-known that HBV-related HCC is a global health problem (78). Studies have found that the hepatitis B virus X protein (HBx), which is a multifunctional protein encoded by the HBx gene, is involved in the metastasis of hepatitis B-related HCC, and since TXNIP overexpression enhances the migration of HepG2 cells, this suggests that HBV-related HCC is mediated by the HBx protein promoting the expression of TXNIP (71). However, it has also been reported that TXNIP overexpression inhibits proliferation and the induction of apoptosis in hepatoma cells by triggering the mitochondrial-mediated production of ROS and activation of the MAPK pathway. It has been suggested that TXNIP could be a new promising drug target for liver cancer (72).

**Table 2:** TXNIP expression in cancers of the digestive system.

**Tumor type**	**Upstream or downstream molecular**	**Significance**	**TXNIP expression**	**Samples**	**References**
Liver cancer	Vitamin D3	VitaminD3 can increase the expression of TXNIP to inhibit the proliferation of liver cancer cells	Down	Cell lines	(69)
Liver cancer	ChREBP	ChREBP binds to the upstream promoter region of TXNIP and promote TXNIP's expression	Down	Cell lines, tissue, animal	(70)
Liver cancer	HBx	X protein promotes the expression of TXNIP	Down	Cell lines, tissue	(71)
Liver cancer	ROS	overexpression of TXNIP inhibits the proliferation of hepatoma cells by producing of ROS	Down	Cell lines, tissue	(72)
Pancreatic cancer	miR-224/HIF1	miR-224 targets the 3'UTR of TXNIP to promote the proliferation and migration of PDAC	Down	Cell lines, tissue	(73)
Pancreatic cancer	Fbw 7	FBW7 can inhibit the expression of c-myc in PDAC to inhibit the occurrence and development of tumor	Down	Cell lines, tissue, animal	(74)
Gastric cancer	TNF-α, NF-κB and COX-2	Disrupting cell growth	Down	Cell lines, tissue	(75)

*ChREBP, the carbohydrate response element binding protein; ROS, Reactive oxygen species; HIF1, hypoxia-inducible factor; Fbw 7, F-box and WD repeat domain-containing7; TNF-α, TumorNecrosisFactor-α; NF-κB, nuclear factor kappa-B; COX-2, Cyclooxygenase-2*.

### TXNIP and Pancreatic Cancer

Pancreatic cancer is a type of digestive tract cancer with a high degree of malignancy and is known to be particularly difficult to diagnose and treat. It is therefore urgent to find new effective treatments because of the extremely high mortality rate for the existing chemotherapy (79). It is well-known that miRNAs. which are non-coding small RNAs, can be used as biomarkers for a tumor's prognosis and could potentially be effective for the treatment of a variety of tumors (80–83). Due to a limited choice of treatment methods, pancreatic ductal adenocarcinoma (PDAC) is the main cause of pancreatic cancer-specific deaths (84). In PDAC, miR-224 targets the 3′UTR of TXNIP and downregulates it, leading to the nuclear translocation of HIF1α, which upregulates HIF1α and in turn promotes the proliferation and migration of PDAC (Figure 2B) (73). Most patients with pancreatic cancer suffer from glucose intolerance or even diabetes. Researchers have shown that the resulting high levels of glucose promote TXNIP expression by activating the p38 MAPK and ERK pathways, thereby promoting tumor development (85). The F-box and WD repeat domain 7 (FBW7) protein is a substrate recognition component of the SKP1-Cul1-F-box (SCF) ubiquitin ligase complex. Increased expression levels of FBW7 can inhibit the expression of c-myc in PDAC, and as a result of the decreased c-myc levels the activity of the TXNIP promoter is increased and TXNIP protein levels increase (Figure 2B). This results in a reduction in glucose metabolism in tumor cells inhibiting tumor occurrence and development (74). Some researchers have shown that in pancreatic cancer, FBW7 regulates the nucleoside transporter protein (ENT1) at the protein level rather than at the transcriptional level to promote chemosensitivity to gemcitabine and may therefore be a feasible target for improving the efficacy of chemotherapy in pancreatic cancer (86).

### TXNIP and Gastroesophageal Cancer

Esophageal cancer is a common gastrointestinal tumor, and the incidence of esophageal adenocarcinoma (EAC) is increasing year by year (87). Despite great efforts to improve the treatment of esophageal adenocarcinoma, its 5-year survival rate is still <20% (88, 89). In EAC, TXNIP has been confirmed to be an independent prognostic factor for distant metastasis-free survival (90). Studies have indicated that TXNIP overexpression can decrease tumor growth in mice. Furthermore, the upregulation of TXNIP, caused by a histone deacetylase inhibitor, can enhance cisplatin-induced apoptosis and DNA damage. Studies have also confirmed that TXNIP overexpression can inhibit the proliferation of esophageal cancer cells and increase the therapeutic sensitivity of cisplatin and other chemotherapy drugs used for the treatment of esophageal cancer (91). The incidence rate of gastric cancer (92) ranks first among malignant tumors in China with the incidence rate in male being about two times higher than that in women (93, 94). Gastric cancer has also been found in the areas of the gastric antrum, the gastric curvature, and the anterior and posterior walls (95–97), with most patients having an adenocarcinoma. Knockout of TXNIP can promote the development of gastric cancer induced by *Helicobacter pylori* in C57BL/6 mice by inhibiting the induction of tumor necrosis factor (TNF-α), nuclear factor kappa-B (NF-κB), and cyclooxygenase-2 (COX-2) (75).

## Cancers Of the Respiratory System and Others (Table 3)

### TXNIP and Lung Cancer

Lung cancer is a type of malignant tumor with the fastest-growing incidence and mortality rates around the world (107). Non-small cell lung cancer (NSCLC) accounts for about 85% of all types of lung cancer and is usually diagnosed during the disease's progression or metastasis phase, so the 5-year survival rate is extremely low (108, 109). For these patients, the treatment strategy is mainly cisplatin (CDDP)-based chemotherapy (110). However, some patients eventually develop resistance to CDDP, which limits its clinical application (111). Therefore, finding new therapeutic targets for lung cancer patients is of great importance. Studies have shown that cisplatin combined with gemcitabine has a coordinative inhibitory effect in cisplatin-resistant cells. In A549 cells, the combination of gemcitabine and cisplatin results in G0/G1 phase arrest and the upregulation of TXNIP. The cytotoxic effect of a TXNIP agonist combined with cisplatin on drug-resistant cells is additive compared to cisplatin alone. Therefore, the increase in TXNIP expression and G0/G1 phase arrest both play key roles in reversing CDDP resistance (112). Researchers have reported that TXNIP is expressed at low levels in NSCLC (113). Yan Li and other researchers have found that hypoxia is a key feature in the tumor microenvironment. Under hypoxic conditions, the level of TXNIP in NSCLC tissues is upregulated, and high expression levels of TXNIP may be a poor prognostic indicator (114). It has been shown that using tyrosine kinase inhibitor (TKIs) to inhibit PI3K/Akt signaling in NSCLC cell lines can lead to a decrease in cell membrane-localized GLUT1, but increases in TXNIP expression in lung cancer tissues (98). Studies have also shown that mTOR and HDAC inhibitors act by converging on the TXNIP antioxidant pathway to co-affect chromatin and transcription, resulting in the death of aggressive neurological malignancies and tumor shrinkage. The mechanism for this is that TXNIP triggers cell death by inhibiting thioredoxin and activating apoptotic signal-regulated kinase 1 (ASK1) signaling. This drug combination can also kill mutant NSCLC and may therefore be important in the treatment of NSCLC (99). Sodium butyrate (NaBu) and sodium 4-phenylbutyrate (4PBA) have broad prospects for the treatment of cancer. In lung cancer cells, TXNIP regulates NaBu- instead of 4PBA-induced H4K5 acetylation and H3K4 trimethylation by increasing the expression of WDR5 after which there is an increase in the activation of caspase 3/7 and cell death (Figure 2D) (100). TNF receptor-related factor 6 (TRAF6) promotes the development of lung cancer by bridging the NF-κB and RAS pathways. Some researchers have found that NaBu-induced TXNIP can interact with TRAF6 through its PPxY motif, which can cause changes in TXNIP expression and its polyubiquitination, which then affect tumor migration and proliferation in NSCLC (101). Several studies have shown that D-allose, a rare sugar, can inhibit the growth of many malignancies. D-allose can increase TXNIP expression at both protein and mRNA levels, thereby inhibiting the growth of NSCLC (115). The micro RNA miR-411-5p/3p is significantly upregulated in human NSCLC tissues and cell lines, and the overexpression of miR-411-5p/3p can inhibit the expression of SPRY4 and TXNIP, which in turn promote tumor proliferation and migration, preventing apoptosis in NSCLC cell lines (102).

**Table 3:** TXNIP expression in cancers of the respiratory system and others.

**Tumor type**	**Upstream or downstream molecular**	**Significance**	**TXNIP expression**	**Samples**	**References**
Lung cancer	PI3K/Akt	(TKIS) inhibits PI3K/Akt signaling in NSCLC's cell lines to increase the expression of TXNIP	Down	Cell lines	(98)
Lung cancer	ASK1	The drug combination can kill mutant non-small cell lung cancer	Down	Cell lines, tissue	(99)
Lung cancer	WDR5	TXNIP increase WDR5 expression to increase lung cell death	Down	Cell lines	(100)
Lung cancer	TRAF6	TXNIP can interact with TRAF6 to affect NSCLC's migration and proliferation	Down	Cell lines	(101)
Lung cancer	miR-411-5p/3p	Overexpression of miR-411-5p/3p can inhibit the expression of SPRY4 and TXNIP to promotes tumor's proliferation and migration	Down	Cell lines, tissue	(102)
Leukemia	ChREBP	ChREBP promotes the development of ROS to promote the occurrence and development of AML	Down	Cell lines, tissue	(103)
Leukemia	JQ1	JQ1 can reduce the expression of myc to activate the ASK1-MAPK pathway, which leads to the death of AML cells	Down	Cell lines, tissue, animal	(104)
Osteosarcoma	PRMT5	PRMT5 overexpression might confer resistance to chemotherapy	Down	Cell lines, tissue	(105)
Neuroblastoma	ROS	Fenofibrate inhibited proliferation and migration of NB cells by increasing intracellular ROS and up-regulating TXNIP expression	Down	Cell lines, tissue	(106)

*PI3K, Phosphatidylinositide 3-kinases; Akt-protein kinaseB(PKB); TKIS, tyrosine kinase inhibitors; ASK1, Apoptosis signal-regulating kinase 1; TRAF6-TNF, receptor associated factor 6; JQ1, an Inhibitor of BET bromodomain; PRMT5, protein arginine methyltransferase 5*.

### TXNIP and Leukemia

Acute myeloid leukemia (AML) is a disease which is characterized by clonal proliferation and is derived from primitive hematopoietic stem cells or progenitor cells. It has an extremely high mortality and poor prognosis in elderly patients (116, 117). Cytogenetic analysis revealed that there are a large number of non-random chromosomal abnormalities in AML, with about 60% of adult AML patients being diagnosed with gene abnormalities (118). TXNIP has been shown to be involved in the reactive oxygen-induced stress responses in mouse leukemia. For example, in screening of new disease genes in mouse leukemia caused by a murine leukemia virus (MLV), TXNIP is found to be a common target for MLV integration. High levels of TXNIP also inhibit the proliferation of myeloid progenitor cells, thereby promoting the occurrence and development of AML and MLV-induced mouse leukemia (119). In AML, the histone methyltransferase inhibitor (DZNep) can increase the levels of TXNIP by clearing the polycomb repressive complex 2 (PRC2) protein and inhibiting the expression of the enhancer of zeste homolog 2 (EZH2) gene, thus inducing ROS generation and leading to apoptosis in AML cells (Figure 2D) (120). Studies have also shown that ChREBP can promote the generation of ROS by downregulating TXNIP expression, thereby promoting the occurrence and development of AML (103). In the process of treating AML, overcoming its drug resistance is a key issue. In AML cells treated with fludarabine (FA), toll-like receptor 4 (TLR 4) or TNF-α-mediated activation of the NF-kB signaling pathway can inhibit TXNIP expression, thereby reducing FA-induced cytotoxicity and increasing cell viability (121). Standard therapies for AML rarely achieve a clinical cure (122), and studies have shown that most AML patients do not benefit from existing approved targeted therapies (123). Therefore, finding new therapeutic targets for the treatment of AML is of paramount importance. JQ1 (a BET bromodomain inhibitor) can reduce the expression of c-myc by inhibiting BRD4 (recombinant human bromodomain domain binding protein 4). This reduction in c-myc upregulates TXNIP by inhibiting miR-17-92 and then activates the ASK1-MAPK pathway, which leads to the death of AML cells through the intrinsic apoptotic pathway (Figure 2D) (104).

### TXNIP and Osteosarcoma

Osteosarcoma (OS) is the most common primary malignant bone tumor (124). For patients who are not suitable for surgical treatment, preoperative and postoperative chemotherapy such as CDDP is the standard treatment method. Although neoadjuvant chemotherapy has made significant progress, the prognosis of OS has hardly improved over the past few decades (125). Cell senescence is defined as a permanent state of cell-cycle arrest. A variety of stimuli, such as DNA damage and oxidative stress, have been shown to cause cell senescence (126, 127). Importantly, TXNIP has been shown to be another important regulator of cell senescence (128). Researchers have found that protein arginine methyltransferase 5 (PRMT5) is overexpressed in OS and that PRMT5 plays an important role in regulating cell senescence in OS by affecting the repair of DNA damage. Furthermore, TXNIP is a key factor in the DNA damage and cell aging induced by PRMT5 depletion. Targeting the PRMT5/TRIM21/TXNIP signal transduction pathway could provide new therapies for the treatment of OS (105).

### TXNIP and Neuroblastoma

High-risk neuroblastoma (HR-NB) is a disease which is one of the most difficult childhood cancers to cure (129). Fenofibrate has been reported to play an antitumor role in several human cancers. In particular, it has been found that fenofibrate inhibits the proliferation of NB cells, significantly increasing intracellular ROS levels, upregulating TXNIP expression, and promoting cell apoptosis (106). Researchers have also found that estrogen can protect neuroblastoma cells from amyloid-β42 (Aβ42)-induced apoptosis through the TXNIP/TRX axis and AMPK signaling pathway (130).

## Conclusions

**Figure 3:**
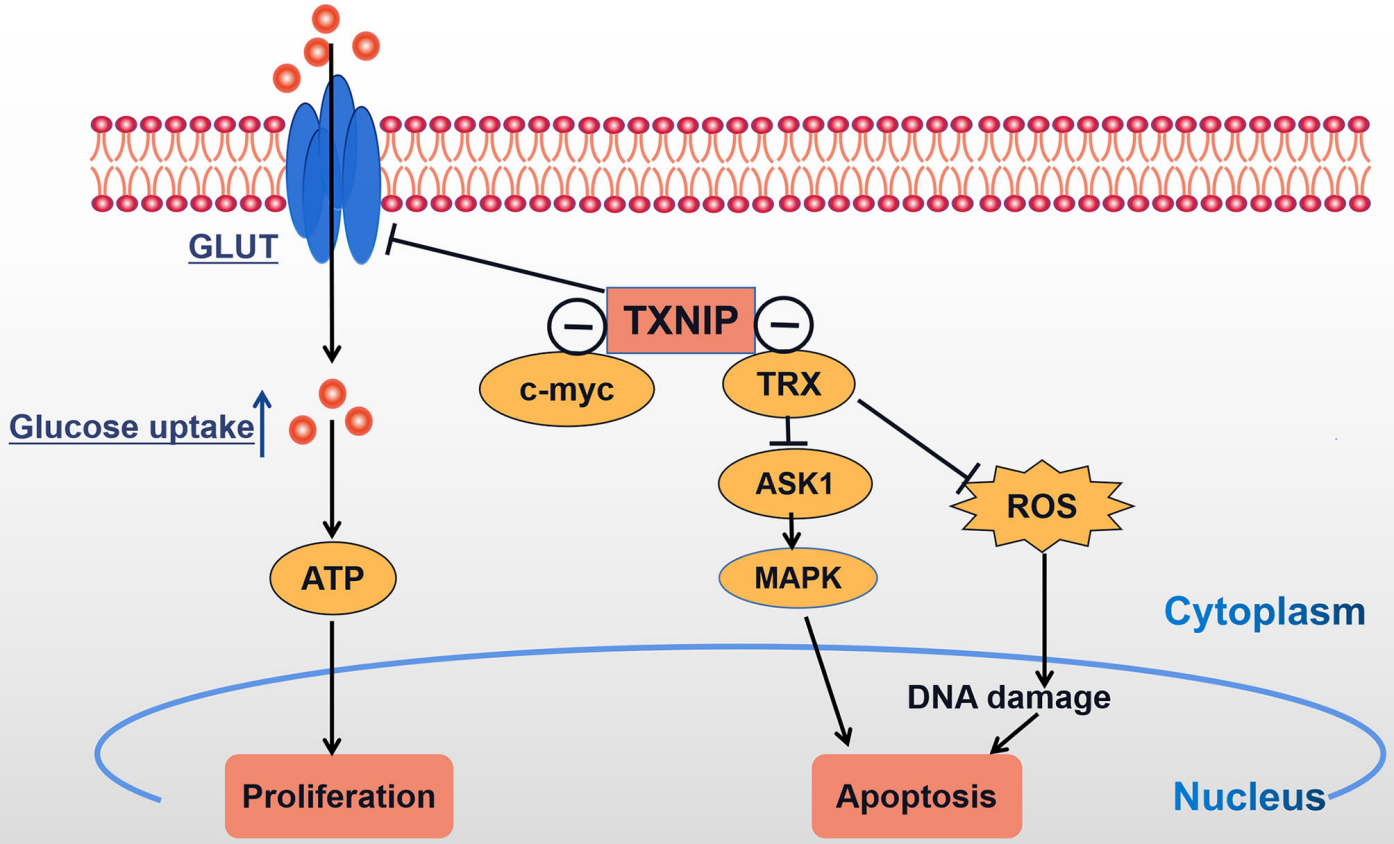
TXNIP acts as a tumor suppressor gene: TXNIP inhibits tumor cell proliferation and promotes tumor cell apoptosis by participating in metabolic reprogramming and oxidative stress; ⊝ signs indicate TXNIP-negative interaction.

With the further study of TXNIP, TXNIP is now expected to become a molecular target for the treatment of different malignancies. TXNIP is expressed at low levels in a variety of malignancies and the overexpression of TXNIP inhibits the proliferation of cancer cells, and so it can be considered as a potential tumor suppressor gene. We conclude that TXNIP acts as a tumor suppressor gene by participating in metabolic reprogramming and oxidative stress (Figure 3). Multiple evidence has shown the abnormal expression and prognostic function of TXNIP in different tumors and illuminated the different molecular mechanisms leading to the acquisition of malignant phenotypes. Given the fact that the morbidity and mortality of cancer in China are continuously rising, seriously threatening people's lives, the prospect of regulating the levels of TXNIP by either genetic means or through drug development gives hope that new tumor treatments can be found.

## Author Contributions

YC is responsible for writing this review. WC, JN, SW, TD, and JJ contributed the same. XF and BZ are responsible for reviewing and revising. All authors contributed to the article and approved the submitted version.

## Conflict of Interest

The authors declare that the research was conducted in the absence of any commercial or financial relationships that could be construed as a potential conflict of interest.

## Abbreviations

TRX: thioredoxin
TXNIP: thioredoxin interacting protein
SH3: src homology domain 3
EMT: epithelial mesenchymal transition
TNBCs: triple negative breast cancers
JAK-STAT: janus kinase/signal transducers and activators of transcription
PTC: papillary thyroid cancer
RCC: renal cell carcinoma
CCRCC: clear cell renal cell carcinoma
BC: bladder cancer
PCA: prostate cancer
p27: tumor protein 27
GLUT1: glucose transporter type 1
HIF1α: hypoxia-inducible factor-1α
MAPK: mitogen-activated protein kinase
UHRF1: ubiquitin-like ringfinger domains 1
TGF-β: transforming growth factor-β
HDAC1: histone deacetylase 1
AMPK: adenosine 5-monophosphate (AMP)-activated protein kinase
cRAPGEF5: circRNA RAPGEF5
ERK: extracellular regulated protein kinases
GLS 1: glutaminase 1
ChREBP: response element binding protein
ChoRE: carbohydrate response element
RNF2: nuclear factor E2-related factor 2
HCC: hepatocellular carcinoma
PDAC: pancreatic ductal adenocarcinoma
EAC: esophageal adenocarcinoma
ChREBP: the carbohydrate response element binding protein
ROS: reactive oxygen species
HIF1: hypoxia-inducible factor
Fbw 7: F-box and WD repeat domain-containing7
TNF-α: TumorNecrosisFactor-α
NF-κB: nuclear factor kappa-B
COX-2: cyclooxygenase-2
NSCLC: non-small cell lung cancer
CDDP: cisplatin
AML: acute myeloid leukemia
PRC2: polycomb repressive complex 2
EZH2: enhancer of zeste homolog 2
OS: osteosarcoma
PI3K: phosphatidylinositide 3-kinases
PKB: Akt-protein kinaseB
TKIS: tyrosine kinase inhibitors
ASK1: apoptosis signal-regulating kinase 1
TRAF6-TNF: receptor associated factor 6
JQ1: an Inhibitor of BET bromodomain
PRMT5: protein arginine methyltransferase 5
HR-NB: high risk neuroblastoma
NB: neuroblastoma.
